# Application of Moran’s Test with an Empirical Bayesian Rate to Leading Health Care Problems in Taiwan in a 7-Year Period (2002–2008)

**DOI:** 10.5539/gjhs.v4n5p63

**Published:** 2012-07-24

**Authors:** Pui-Jen Tsai

**Affiliations:** Center for General Education, Aletheia University, New Taipei, Republic of China (Taiwan)

**Keywords:** local indicators of spatial association, empirical Bayesian rate, logistic regression, prevalence rate, disease map

## Abstract

**Purpose:**

This study focused on using Moran’s tests and logistic regression to detect changes in spatial clustering for females and males.

**Methods:**

For spatial distribution analysis, an average morbidity rate for a 7-year period was calculated. Medical cases from Taiwan National Health Insurance (NHI) were used as the numerator, and the denominator was the average mid-year population. Spatial analysis techniques, with a morbidity-smoothing coefficient estimate based on the empirical Bayesian method, were incorporated and applied to global and local Moran tests. In addition, we used a logistic regression model to test the characteristics of similarity and dissimilarity between males and females and to formulate the common spatial risk.

**Results:**

The mean found by local spatial autocorrelation analysis was used to identify spatial cluster patterns. There is great interest in discovering the relationship between leading health care problems and spatial risk factors. For example, in Taiwan, the geographic distribution of clusters where neoplasms were prevalent was found to closely correspond to the locations in the arseniasis-endemic areas of Southwestern and Northeastern Taiwan, as well as to locations in the Tainan urban area (for females) and clusters in Changhua County and Yunlin County (for males). The high-density populations in urban areas showed carcinogen clusters in Taiwan’s 3 main urban centers (i.e., Taipei, Taichung, and Kaohsiung) for female neoplasms.

**Conclusion:**

Cluster mapping helped clarify issues such as the spatial aspects of both the internal and external correlations for leading health care events. This information greatly assists in assessing spatial risk factors, which facilitates the planning of the most advantageous types of health care policies, as well as the implementation of effective health care services.

## 1. Introduction

Spatial epidemiology is the description and the analysis of geographic variations for diseases with respect to demographic, environmental, behavioral, socioeconomic, genetic, and infectious risk factors (Elliott & Wartenberg, 2004). Common spatial techniques for health research include disease mapping, distance calculations, spatial aggregation, clustering, spatial smoothing and interpolation, identification of risk factors through comparisons, and spatial regression (Gesler, 1986; Werneck, 2008; Auchincloss et al., 2012). All of these methods are useful when assessing risk factors. Spatial clustering techniques are vital for statistical consideration, and form the foundation in the development of models for predicting disease risk sites. Disease risk sites are areas located close to one another that tend to share similar disease risk factors because they share similar environments; they are also often connected by the spread of communicable disease through vectors or host dispersal (Waller & Gotway, 2004).

Measures of morbidity rates of disease patterns in small areas have limitations. Because populations are dynamic, mapping and statistical comparisons using multiple variances must be conducted. In addition, morbidity rates in areas of low population have higher variances and are more unstable than those in high population areas (Moulton et al., 1994; Elliott et al., 2000). Low population areas are often rural and cover large areas, giving unjustified visual impressions that may be unreliable because of higher variances. One method of circumventing this problem is to use “smoothed” estimates of diseases (Olsen et al., 1996), such as the empirical Bayesian (EB) method. The Bayesian approach is a method of statistical estimation where observed data and prior knowledge of the parameters of interest are considered when estimating their values.

Local indicators of spatial association (LISA) detect local spatial autocorrelations in aggregated data by dividing Moran’s statistic into contributions for each area within a study region (Anselin, 1995). A critical method combining both the empirical Bayesian estimate and the LISA statistic has been well-documented in many applications, such as neonatal and post-neonatal mortality (Neto et al., 2001), Burkitt’s lymphoma (Rainey et al., 2007), motorcycle accidents (Erdogan, 2009; Silva et al., 2011), human cryptosporidiosis (Callaghan et al., 2009), and legionellosis (Gómez-Barroso et al., 2011).

This study focuses on using a direct method of Anselin’s Local Moran test with empirical Bayesian rates and logistic regression to detect changes in spatial clustering for females and males. We propose a method for identifying the spatial clustering associated with the 18 leading health care problems based on medical care data collected by Taiwan National Health Insurance (NHI). In addition, we investigate the potential spatial risks that could contribute to these health care events, redefining epidemiologic and spatially referenced data.

## 2. Method

### 2.1 Data Collection and Management

The Taiwan NHI program was initiated in 1995. The coverage rate of the program increased from 92.4% in 1995 to more than 96.2% in 2000, and then increased to 98% following the inclusion of active members of military forces in 2001. When the NHI medical care data were properly collected and analyzed, a medical visiting population according to disease could be used for reference in the calculation of prevalence and incidence of various diseases. In early 1998, the NHI data that were available and were relevant to medical care, such as leading health care problems, were published in relation to sex and age (Department of Health, 2012a). In addition, regional data related to sex were reclassified and reprocessed in relation to smaller units or areas (e.g., precincts or townships rather than the entire country) and announced by the Collaboration Center for Health Information Application (CCHI; Department of Health, 2012b). The smallest administrative units coded for examination of the various disease cases or health care events were precincts and townships. These reports provided an accurate and reliable data source for the investigation of health care issues in Taiwan.

Data were collected from contractual medical care institutions where the NHI covers the costs of prescription medicines and treatment at outpatient clinics. These types of facilities accumulate detailed databases on medical costs for inpatient care. The number of outpatient cases was classified in accordance to disease codes, as defined in the 1975 edition of The International Classification of Diseases, 9th Revision, Clinical Modification (ICD 9 CM). Patients suffering from diseases that were difficult to classify into a specific code or who had mismatched ID numbers were not included in the final statistical data set. Disease codes were classified according to sex and age. Cases with the same ID numbers, but that exhibited different diseases, were counted as different instances. The age-adjusted standard prevalence rates were calculated with a direct adjustment using the global population in 2000 as the standard population (Ahmad et al., 2001). The age-adjusted standard prevalence rates during 2002–2008 were calculated according to the 7-year prevalence rates that were weighted by people each year. The results elucidated the leading health care problems for males and females in Taiwan.

Medical care data obtained from the 2002–2008 NHI reports were examined, and the morbidity rates of the 18 leading health care problems were calculated. Disease classifications (according to the ICD 9 CM) included the following (disease codes are indicated within parentheses): infectious and parasitic diseases (ICD 01-07); neoplasms (ICD 08-17); endocrine, nutritional, and metabolic diseases and immunity disorders (ICD 18-19); diseases of the blood and blood-forming organs (ICD 20); mental disorders (ICD 21); diseases of the nervous system and sense organs (ICD 22-24); diseases of the circulatory system (ICD 25-30); diseases of the respiratory system (ICD 31-32); diseases of the digestive system (ICD 33-34); diseases of the genitourinary system (ICD 35-37); diseases of the skin and subcutaneous tissue (ICD 42); diseases of the musculoskeletal system and connective tissue (ICD 43); congenital anomalies (ICD 44); certain conditions originating in the perinatal period (ICD 45); signs, symptoms, and ill-defined conditions (ICD 46); injury and poisoning (ICD 47-56, E47-56); other reasons for contact with health services (V0); and complications of pregnancy, childbirth, and the puerperium (ICD 38-41).

### 2.2 Study Area for Spatial Autocorrelation Calculations

**Figure 1:**
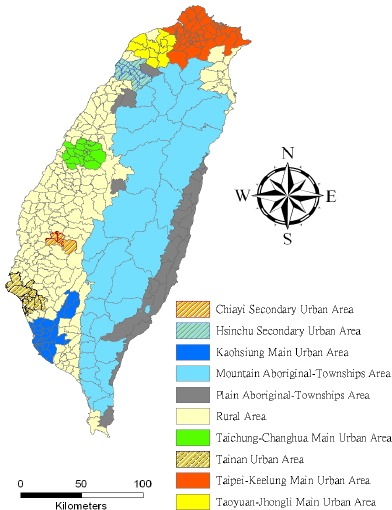
Map of urban areas and aboriginal townships in the study area Map of the study area divided into 348 administrative districts, including seven urban areas and an integrated area of 54 aboriginal townships located in the plains and mountains.

The study area included the main island of Taiwan (excluding all surrounding islets) that, in the year 2000, had more than 22 million inhabitants living in an area of 36,000 km^2^. This area that was assessed totaled 348 local administrative government areas, including five main urban areas, two secondary urban areas, 162 rural townships, and 54 aboriginal townships located in the plains or in mountainous regions (Fig. 1). According to a 2002 Ministry of the Interior report, urban areas are classified as regions having at least one metropolitan center, and they can include neighboring cities and townships that share socioeconomic activities. The main urban areas were defined in this study as those with a population larger than 1 million, specifically, Taipei-Keelung, Kaohsiung, Taichung-Changhua, Jhongli-Taoyuan, and Tainan. Secondary urban areas were defined as those with a residential population ranging from 0.3 to 1 million (e.g., Hsinchu and Chiayi).

### 2.3 Empirical Bayesian Smoothing Rates

The raw rates were derived from various precincts and townships across a region, and may have resulted in unstable rates because of the small number of cases from counties with a small population base. The corollary to this is that the rates may not fully represent the relative magnitude of the underlying risks if they are compared with other counties with a high population base. To reduce this discrepancy, empirical Bayesian smoothing, which was proposed by Clayton and Kaldor (1987), was applied to the computed raw rates. The formula for the empirical Bayesian smoothing is Ŕ = μ + ś(r - μ), where Ŕ is the new smoothed rate estimate, μ is the global population-weighted mean, ś is the shrinkage factor, and r is the level of incidence rate (Waller & Gotway, 2004). In this study, we calculated the Bayesian smoothing rate by using the averaged visiting medical cases during 2002–2008 as the numerator, and the average population in each township was the denominator (Department of Health, 2012b).

### 2.4 Global Moran’s Statistic

The global Moran’s spatial autocorrelation was used to assess the correlation among neighboring observations and to identify patterns and levels of spatial clustering in neighboring districts (Boots & Getis, 1998). Moran’s statistic, similar to the Pearson correlation coefficient (Cliff & Ord, 1973), was calculated using the following formula:


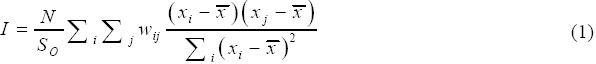


where N is the number of districts, w_ij_ is the element in the spatial weight matrix corresponding to the observation pair **i**, **j** and **x_i_** and **x_j_** observations for the areas i and j with the mean, x̄ and


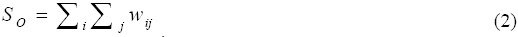


Because the weights were row-standardized (∑w_ij_ = 1), the first step in spatial autocorrelation analysis was to construct a spatial weight matrix that contained information on the neighborhood structure for each location. Adjacency was defined as immediately neighboring administrative districts, including the district itself. Non-neighboring administrative districts were assigned a weight of zero.

Spatial contiguity for polygons was defined as the property of sharing a common boundary or vertex. Contiguity analysis is an important method for assessing unusual features in connectivity distribution (P. Legendre & L. Legendre, 1998; Grubesic, 2008). The Queen’s measure of contiguity can be used to compensate for spatial contiguity by incorporating both the Rook and Bishop relationships into a single measure (Grubesic, 2008). The administrative districts considered in this study were highly irregular in both shape and size. The most appropriate method was the first-order queen polygon contiguity method for quantifying the spatial weights matrix for the analysis of connectivity (Tsai et al., 2009). Based on this approach, the spatial weight/connectivity matrices were determined and used in conjunction with the global Moran’s statistic and the LISA calculations described below.

Moran’s I values may range from -1 (dispersed) to +1 (clustered). A Moran’s I value of 0 suggests complete spatial randomness. A random permutation procedure recalculates a statistic many times by reshuffling the data values among the map units to generate a reference distribution. The obtained calculated statistic, based on the observed spatial pattern, is then compared to the reference distribution, and a pseudo significance level (pseudo *p* value) is computed. To verify that the value of Moran’s I significantly differed from the expected value, we used a Monte Carlo randomization test with 9,999 permutations to achieve significant values. The data values were reassigned among the N locations, providing a randomized distribution against which the observed value could be judged. If the observed value of I was within the tails of this distribution, there was a significant spatial autocorrelation in the data, and there was a pseudo *p* value smaller than 0.05, and the assumption of independence among the observations could be rejected (Cliff & Ord, 1981).

### 2.5 Local Indicators of Spatial Association (LISA) Statistic

The LISA statistic provides information related to the location of spatial clusters and outliers and the types of spatial correlation. Local statistics are important because the magnitude of the spatial autocorrelation was not necessarily uniform over the study area (Anselin, 1995; Ord & Getis, 1995). LISA divided the study area into smaller locations, enabling the assessment of significant local spatial clustering around an individual location. In addition to the degree of spatial clustering, detailed variations of clustering in the locally defined geo-space were identified, as well as the locations of the spatial clusters. The local version of Moran’s at location **i** is given by


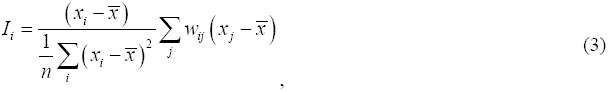


where n indicates the total number of locations (348 townships); x_i_ denotes the value of the variable of interest, X at location I; x_j_ denotes the observation at neighboring locations j, and x̄ is the sample average of X. The notation w_ij_ was the spatial weight matrix, which defined spatial interaction across the study regions. In general, w_ij_ = 1 if location i and location j were neighboring (shared a common boundary); otherwise, w_ij_= 0. In this study, spatial contiguity was assessed as the first order queen’s contiguity that defines spatial neighbors as those areas with shared borders and vertexes.

Significance was tested by comparison to a reference distribution obtained by random permutations (Anselin, 1995). This analysis used 9,999 permutations to determine the differences among the spatial units. A positive value for the local Moran’s I index (*I_i_*) indicates that a feature has neighboring features that have similarly high or low attribute values, meaning that it is a part of a cluster. A negative value for (*I_i_*) indicates that a feature has neighboring features that have dissimilar values, indicating that this feature is an outlier. In either circumstance, the *p* value for the feature must be small enough for the cluster or outlier to be considered statistically significant. LISA enables distinctions to be made among a statistically significant (0.05 level) cluster of high values (HH), a cluster of low values (LL), an outlier in which a high value is surrounded mostly by low values (HL), and an outlier in which a low value is surrounded mostly by high values (LH). In addition, for the value of a z-score larger than +1.96, the outcomes are defined as clusters with both (HH) and (LL). If the value of a z-score is less than -1.96, the outliers are considered clusters with (HL) and (LH).

### 2.6 Spatial Similarity Comparison Using Logistic Regression Analysis

In addition to mapping, similarities between the spatial distribution patterns for males and females were determined using logistic regression analysis. The binary response indicates whether significant autocorrelation existed between administrative districts or areas. The correlation is better (higher) if the value of the z-score of the local Moran’s I statistic is larger than +1.96 (clusters with hot spots and cold spots); otherwise, it is deemed low. The model is expressed as


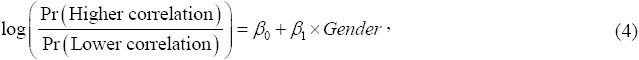


where sex is considered an explanatory variable in the logistic regression model, and the two β values were the logistic regression coefficients of the model. Pr (Higher correlation) and Pr (Lower correlation) denote the higher and lower correlation probabilities, respectively.

Modeling of logistic regression was performed using SPSS 12. The global and local Moran’s statistics with empirical Bayesian rates were calculated using Geoda095i (http://www.geoda.uiuc.edu/), an open source spatial analysis system, and visualized on LISA cluster maps using ArcMap 9.3.

## 3. Results

A summary of the results of the prevalence rates for the 18 health care problems during 1998–2008 in Taiwan is shown in Table 1. The results showed that the 18 prevalence rates related to the leading health care problems for females were higher than those for males. Sex ratios, defined as the ratio of females to males, typically ranged from one to three, although they were occasionally higher than three (e.g., for diseases of the genitourinary system).

**Table 1:** Prevalence rates of the 18 leading health care problems in Taiwan by gender, during 1998–2008

Leading health care problems (ICD code)	female*	male*	ratio (female:male)
Infectious and parasitic diseases (ICD 01-07)	18782	17315	1.08
Neoplasms (ICD 08-17)	7160	3434	2.08
Endocrine, nutritional and metabolic diseases and immunity disorders (ICD 18-19)	11195	10297	1.09
Diseases of the blood and blood-forming organs (ICD 20)	2339	979	2.39
Mental disorders (ICD 21)	8687	6226	1.40
Diseases of the nervous system and sense organs (ICD 22-24)	39468	31357	1.26
Diseases of the circulatory system (ICD 25-30)	14040	12765	1.10
Diseases of the respiratory system (ICD 31-32)	77604	68684	1.13
Diseases of the digestive system (ICD 33-34)	64183	56426	1.14
Diseases of the genitourinary system (ICD 35-37)	31879	8972	3.55
Diseases of the skin and subcutaneous tissue (ICD 42)	33896	27008	1.26
Diseases of the musculoskeletal system and connective tissue (ICD 43)	28359	22972	1.23
Congenital anomalies (ICD 44)	1022	937	1.09
Certain conditions originating in the perinatal period (ICD 45)	390	226	1.72
Signs, symptoms and ill-defined conditions (ICD 46)	38544	27865	1.38
Injury and poisoning (ICD 47-56, E47-56)	25576	26444	0.97
Other reasons for contact with health services (V0)	24141	12263	1.97
Complications of pregnancy, childbirth and the puerperium (ICD 38-41)	3202	n.d.	n.d.

* indicates age-adjusted prevalence rates per 100,000 people n.d. represents no detection

Table 2 shows a summary of the results from the global autocorrelation statistics for the 18 health care problems according to sex. The results of the global Moran’s test for the majority of the cases related to the leading health care problems were statistically significant, having a pseudo *p* value smaller than .05 and indicating spatial heterogeneity. However, contrasting results (a pseudo *p* value greater than .05) emerged in six cases, which were related to diseases of the circulatory system for females, diseases of the musculoskeletal system and connective tissue for females and males, injury and poisoning for females and males, and other reasons for contacting health services for females.

**Table 2:** Global autocorrelation analysis of data for the 18 leading health care problems in Taiwan, according to gender, during 2002–2008

Leading health care problems (ICD code)	Female		Male	

	Moran’s I	p-value^a^	Moran’s I	p-value^a^
Infectious and parasitic diseases (ICD 01-07)	0.34	0.0001	0.36	0.0001
Neoplasms (ICD 08-17)	0.71	0.0001	0.66	0.0001
Endocrine, nutritional and metabolic diseases and immunity disorders (ICD 18-19)	0.60	0.0002	0.54	0.0137
Diseases of the blood and blood-forming organs (ICD 20)	0.46	0.0001	0.45	0.0001
Mental disorders (ICD 21)	0.49	0.0001	0.43	0.0001
Diseases of the nervous system and sense organs (ICD 22-24)	0.63	0.0001	0.61	0.0001
Diseases of the circulatory system (ICD 25-30)	0.70	0.9985	0.56	0.0001
Diseases of the respiratory system (ICD 31-32)	0.42	0.0001	0.53	0.0001
Diseases of the digestive system (ICD 33-34)	0.64	0.0001	0.62	0.0001
Diseases of the genitourinary system (ICD 35-37)	0.45	0.0001	0.46	0.0001
Diseases of the skin and subcutaneous tissue (ICD 42)	0.46	0.0001	0.41	0.0001
Diseases of the musculoskeletal system and connective tissue (ICD 43)	0.73	0.9968	0.67	0.9966
Congenital anomalies (ICD 44)	0.51	0.0001	0.58	0.0001
Certain conditions originating in the perinatal period (ICD 45)	0.62	0.0001	0.64	0.0001
Signs, symptoms and ill-defined conditions (ICD 46)	0.69	0.0001	0.65	0.0001
Injury and poisoning (ICD 47-56, E47-56)	0.57	0.2056	0.59	0.0691
Other reasons for contact with health services (V0)	0.68	0.5394	0.65	0.0014
Complications of pregnancy, childbirth and the puerperium (ICD 38-41)	0.47	0.0001	n.d.	n.d.

a indicates a pseudo p-value at permutation of 9999

n.d. represents no detection

**Figure 2:**
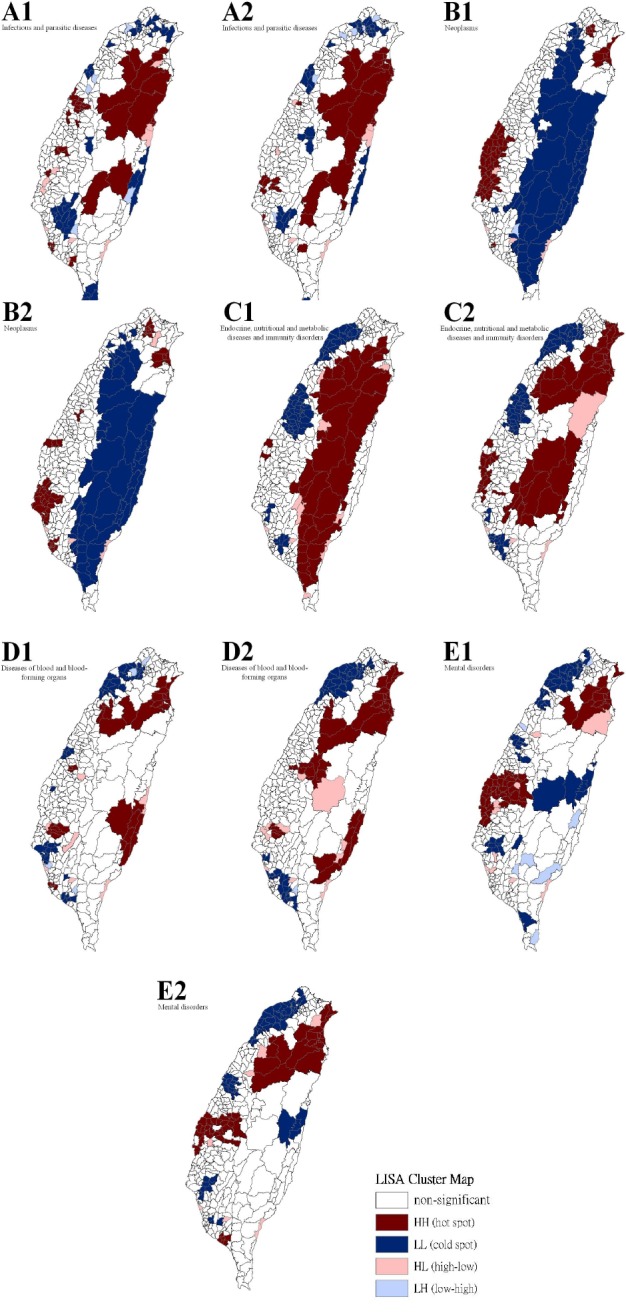
Empirical Bayesian smoothed spatial clusters of the 18 leading health care problems from the order 1 to 5 in Taiwan

**Figure 3:**
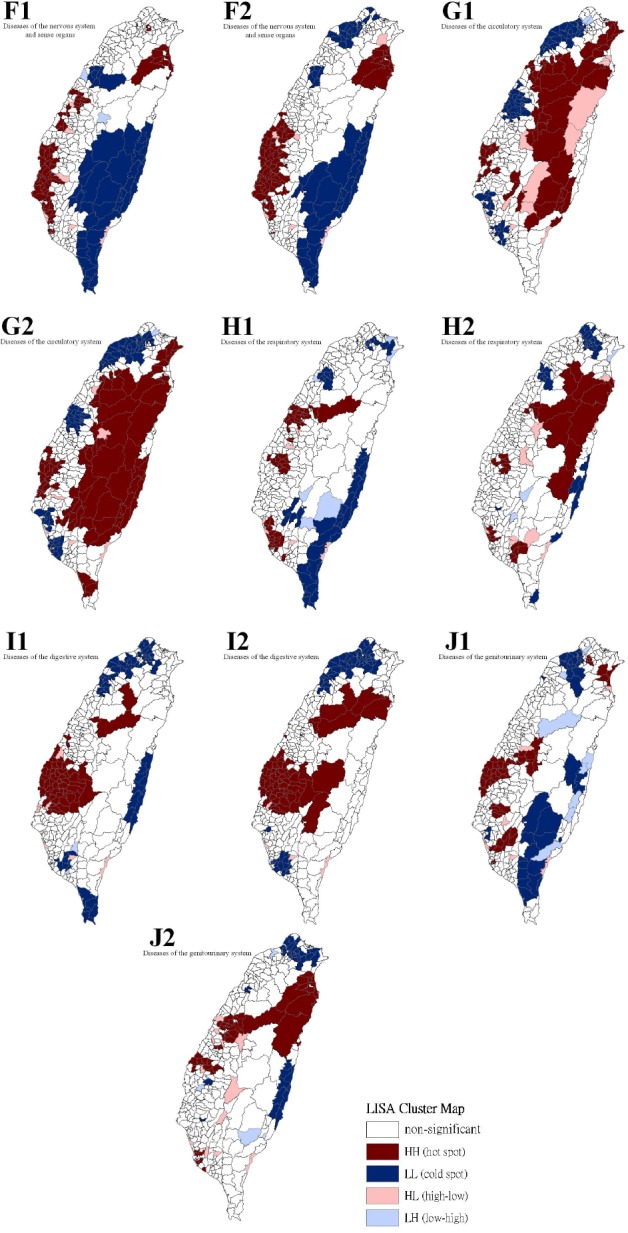
Empirical Bayesian smoothed spatial clusters of the 18 leading health care problems from the order 6 to 10 in Taiwan

**Figure 4:**
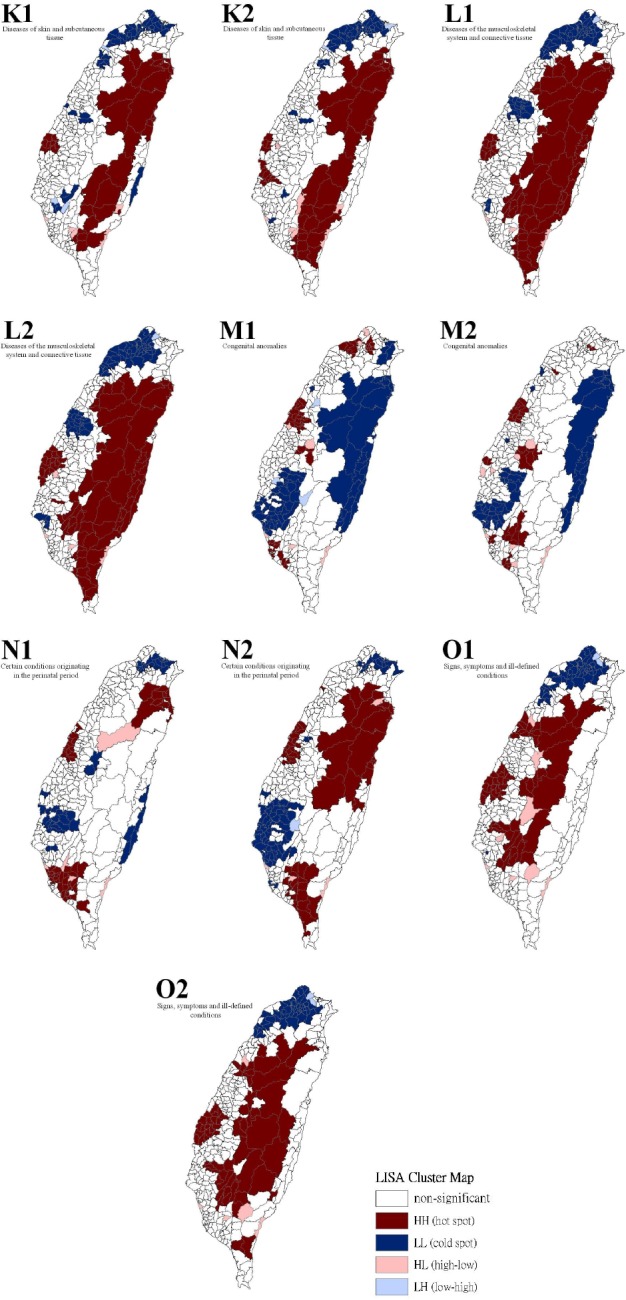
Empirical Bayesian smoothed spatial clusters of the 18 leading health care problems from the order 11 to 15 in Taiwan

**Figure 5:**
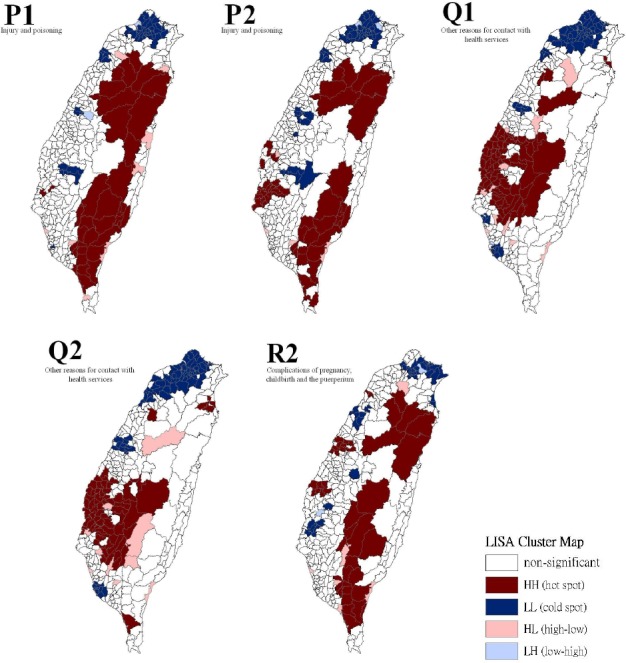
Empirical Bayesian smoothed spatial clusters of the 18 leading health care problems from the order 16 to 18 in Taiwan

Table 3 show a summary of the typology patterns (as calculated using a LISA statistic) that were categorized as clusters or non-clusters at a z-score larger than +1.96, indicating statistical significance (.05 level). Table 3 also shows a comparison of the 17 health care problems shared by gender. No statistically significant dissimilarity (*p* > .05) was observed between the spatial distribution patterns for males and females for 13 of the 17 spatial clusters. Dissimilarities were found for the leading health care problems for diseases of the circulatory system, diseases of the respiratory system, congenital anomalies, and conditions originating in the perinatal period. Figures 2 to 5 show the spatial clusters—obtained using the LISA statistic—for the 18 leading health care problems for males and females in Taiwan during 2002–2008.

**Table 3:** Logistic regression model comparisons of the 17 leading health care problems in Taiwan by gender, 2002–2008

Leading health care problems (ICD code)	p-value	description
Infectious and parasitic diseases (ICD 01-07)	0.578	similarity^a^
Neoplasms (ICD 08-17)	0.691	similarity^a^
Endocrine, nutritional and metabolic diseases and immunity disorders (ICD 18-19)	0.179	similarity^a^
Diseases of the blood and blood-forming organs (ICD 20)	0.605	similarity^a^
Mental disorders (ICD 21)	0.369	similarity^a^
Diseases of the nervous system and sense organs (ICD 22-24)	0.43	similarity^a^
Diseases of the circulatory system (ICD 25-30)	0.022	dissimilarity
Diseases of the respiratory system (ICD 31-32)	0.007	dissimilarity^a^
Diseases of the digestive system (ICD 33-34)	0.475	similarity^a^
Diseases of the genitourinary system (ICD 35-37)	0.435	similarity^a^
Diseases of the skin and subcutaneous tissue (ICD 42)	0.299	similarity^a^
Diseases of the musculoskeletal system and connective tissue (ICD 43)	0.251	similarity
Congenital anomalies (ICD 44)	0.03	dissimilarity^a^
Certain conditions originating in the perinatal period (ICD 45)	0.001	dissimilarity^a^
Signs, symptoms and ill-defined conditions (ICD 46)	0.747	similarity^a^
Injury and poisoning (ICD 47-56, E47-56)	0.152	similarity
Other reasons for contact with health services (V0)	0.878	similarity

a indicates a comparison of both males and females for which all of Moran’s test results are clusters (results based on Table 2)

Maps showing the spatial clusters of the 18 leading health care problems in Taiwan: infectious and parasitic diseases are designated by A; neoplasms, B; endocrine, nutritional and metabolic diseases and immunity disorders, C; diseases of the blood and blood-forming organs, D; mental disorders, E.

Sex is indicated by number, where males are 1 and females are 2.

Maps showing the spatial clusters of the 18 leading health care problems in Taiwan: diseases of the nervous system and sense organs are designated by F; diseases of the circulatory system, G; diseases of the respiratory system, H; diseases of the digestive system, I; diseases of the genitourinary system, J.

Sex is indicated by number, where males are 1 and females are 2.

Maps showing the spatial clusters of the 18 leading health care problems in Taiwan: diseases of the skin and subcutaneous tissue are designated by K; diseases of the musculoskeletal system and connective tissue, L; congenital anomalies, M; certain conditions originating in the perinatal period, N; signs, symptoms, and ill-defined conditions, O.

Sex is indicated by number, where males are 1 and females are 2.

Maps showing the spatial clusters of the 18 leading health care problems in Taiwan: injury and poisoning are designated by P; other reasons for contacting health services, Q; complications of pregnancy, childbirth and the puerperium, R.

Sex is indicated by number, where males are 1 and females are 2.

## 4. Discussion

Geography is concerned with the identification and explanation of spatial structures, patterns, and processes, as well as with the analysis and explanation of the connections between humans and the environment. The validity of using geography to study disease or health care is based on the appreciation of the factors causing the non-uniformity of disease distribution (Mayer, 1983). These factors include physical and environmental factors, socioeconomic and cultural factors, and genetic factors. For example, disease may be associated with environmental pollution, individual or group behaviors, or associated with a genetic predisposition. Therefore, all of these factors may have spatial distributions that influence the extent and intensity of a particular disease (Moore & Carpenter, 1999). Spatial cluster detection is a vital tool in disease surveillance to identify areas of elevated risk and to generate subsequent hypotheses regarding disease etiology. It also facilitates the planning of health care policies and supports the implementation of effective health care services. The z-scores for the LISA method were calculated using the logistic regression model, and results for the various leading health care problems for females and males were compared. However, the constraint conditions for spatial clustering comparison (such as global Moran’s tested clusters on both sides) had to be satisfied before calculating the logistic regression for the purpose of comparison. Based on this constraint, the results had statistically significant differences for diseases of the respiratory system, congenital anomalies, and certain conditions originating in the perinatal period. An additional 10 cases that were compared were not significantly different. Therefore, the null hypothesis was accepted. The accepted null hypothesis results indicated that common spatial factor(s) may interact with both sexes.

Most of the clusters regarding the 18 leading health care events in Taiwan were discovered in our results, although the risk is poorly understood. Few previous ecological studies relate to health care problems and their correlations to risk factors in Taiwan, although neoplasms and certain conditions originating in the perinatal period have been documented, as discussed below. This assessment of the spatial clustering of Taiwan’s leading health care problems will hopefully contribute to the study of spatial epidemiology.

Residents along the southwestern and northeastern coasts of Taiwan frequently drank well water that was contaminated with a high concentration of arsenic before the establishment of a public water system (Tseng et al., 1961). Residents in these areas had an increased risk of malignant neoplasms, including cancers of the liver, nasal cavity, lungs, skin, bladder and kidneys, for males and for females, as well as prostate cancer in males (Chen & Wang, 1990; Chen et al., 2002). Although well water is no longer used for drinking or cooking following the mid-1970s, there is still a significantly increased risk of urinary cancers (Chiou et al., 2001; Liao et al., 2009) and lung cancer (Chen et al., 2004; Liao et al., 2009) in the arseniasis-endemic areas of Southwestern and Northeastern Taiwan. In addition, Yang and Hsieh (1998) reported that variations in cancer incidences and mortality rates across urbanization gradients were found to have higher rates in urban populations than in rural areas during 1982–1991. Significantly increasing trends associated with increasing urbanization were observed in mortality rates for cancers of the lung, pancreas, and kidneys for both men and women, as well as for male prostate cancer and female breast and ovarian cancer (Yang & Hsieh, 1998). Our results showed clusters for neoplasms in the arseniasis-endemic areas of Southwestern and Northeastern Taiwan, consistent with the results of previous studies that demonstrated that arsenic toxicity has long-term effects. In addition, we identified locations in the Tainan urban area (in females) and clusters in Changhua county and Yunlin county (in males), although the specific spatial risk remains unclear. High-density populations in urban areas showed carcinogen clusters in Taiwan’s three largest cities (Taipei, Taichung, and Kaohsiung) for female neoplasms, but the male population did not show the same clusters in the same locations in Taipei. Further investigation is warranted.

Tsai et al. (2009) reported a cluster pattern for certain disease conditions originating in the perinatal period. Their observations revealed clusters in the central coast of Taiwan for females and males (Tsai et al., 2009). Additional evidence on LISA maps where found for the northern and central mountain regions of Taiwan, and clusters were observed in Southern Taiwan, especially in Pingtung County, as hot spots for females. In addition, for males, the most significant hot spots were in the northeastern region of Taiwan’s Yilan County and locations in Southern Taiwan’s Kaohsiung urban area.

## 5. Conclusion

The method of combining empirical Bayesian smoothing and the LISA statistic was an effective tool for the detection of spatial patterns with raw morbidity rates. Similarity resulted from unchangeable conditions in disease risks. Conversely, dissimilarity was deemed a significant difference of morbidity risks between sexes. This enables planners to assess spatial risk factors and to determine the most advantageous types of health care policies for the planning and implementation of health care services.
